# (+)-*Erythro*-Δ^8′^-7*S*,8*R*-dihydroxy-3,3′,5′-trimethoxy-8-*O*-4′-neolignan, an Anti-Acne Component in Degreasing *Myristica fragrans* Houtt

**DOI:** 10.3390/molecules25194563

**Published:** 2020-10-06

**Authors:** Chia-Jung Lee, Chun-Wei Huang, Lih-Geeng Chen, Ching-Chiung Wang

**Affiliations:** 1 PhD Program in Clinical Drug Development of Herbal Medicine, College of Pharmacy, Taipei Medical University, Taipei 110, Taiwan; cjlee@tmu.edu.tw; 2 Graduate Institute of Pharmacognosy Science, College of Pharmacy, Taipei Medical University, Taipei 110, Taiwan; weiwei19851124@hotmail.com; 3 Traditional Herbal Medicine Research Center, Taipei Medical University Hospital, Taipei 110, Taiwan; 4 Graduate Institute of Biomedical and Biopharmaceutical Sciences, College of Life Sciences, National Chiayi University, Chiayi 600, Taiwan; lgchen@mail.ncyu.edu.tw; 5 School of Pharmacy, Taipei Medical University, Taipei 110, Taiwan

**Keywords:** *Myristica fragrans* Houtt, *Cutibacterium acnes*, *Staphylococcus aureus*, skin irritation, anti-inflammatory effect, anti-acne effect

## Abstract

Acne is a common skin condition observed in adolescents. Nutmeg (*Myristica fragrans* Houtt) (MF) is a well-known traditional Chinese medicine; its major toxic components, safrole and myristicin, are rich in essential oils. Essential oils of MF (MFO) were extracted by hydrodistillation; the residue was extracted using 50% methanol (MFE-M). The minimum inhibitory concentration (MIC) of MFE-M against *Cutibacterium acnes* and *Staphylococcus aureus* was 0.64 mg. Four compounds were obtained from MFE-M: myristicin (**1**), (+)-*erythro*-Δ^8′^-7*S*,8*R*- dihydroxy-3,3,5′-trimethoxy-8-*O*-4′-neolignan (**2**), (+)-*erythro-*Δ^8’^-7-hydroxy-3,4,3’,5’-tetramethoxy 8-*O*-4-neolignan (**3**), and e*rythro*-Δ^8′^-7-acetoxy-3,4,3′,5′-tetramethoxy-8-*O*-4′-neolignan (**4**). Compound **2** exerted the strongest antimicrobial activity, with MICs of 6.25 and 3.12 μg/mL against *C. acnes* and *S. aureus*, respectively. Moreover, **2** inhibited NO, PGE_2_, iNOS, and COX-2 levels in RAW 264.7 cells induced by LPS or heat-killed *C. acnes*; NO production at 50% inhibitory concentrations (IC_50_) was 11.07 and 11.53 μg/mL, respectively. Myristicin and safrole content was higher in MFO than in MFE-M. MFO and MFE-M caused no skin irritation after a single topical application in Wistar rats. MFE-M, with low safrole and myristicin content, did not cause skin irritation and exhibited an anti-acne effect; moreover, **2** was identified as the active substance. Therefore, MFE-M could be employed to develop anti-acne compounds for use in cosmetics.

## 1. Introduction

Acne, a high incidence of which is observed during adolescence, is a long-term skin disease. Typical features of the condition include blackheads or whiteheads, papules, pimples, nodules, and cysts. Acne can appear on the back, chest, neck, shoulders, upper arms, and buttocks. Acne not only causes spots and scars but also causes feelings of inferiority and depression. The following four major factors are the cause of acne formation: (1) hyperkeratosis of the skin, (2) overactive oil glands, (3) bacterial infection (*Cutibacterium acnes* (*previously Propionibacterium acnes*) and *Staphylococcus aureus*), and (4) skin inflammation [1]. Depending on the cause of acne, its clinical treatment involves the following: (1) Use of vitamin A acid (tretionin), salicylic acid, and azelaic acid to abnormal keratinized hair follicle sebocytes; (2) use of hormonal therapy to reduce sebaceous gland activity and oil secretion; (3) use of antibiotics such as clindamycin, erythromycin, and tetracycline to inhibit pathogenic bacteria in hair follicles, and (4) use of NSAIDs to reduce inflammatory responses [2]. However, different therapeutic agents result in skin depigmentation, irritation, and desquamation. Therefore, discovery of a drug that has multiple therapeutic effects, which does not result in skin irritation, is important for the treatment of acne [3].

Nutmeg (*Myristica fragrans*) is a condiment that is well known for the flavor it adds to food. Additionally, it is a famous traditional Chinese medicine. *M. fragrans* (MF) is widely cultivated in Asia, including Indonesia, Thailand, Japan, and China. Traditional functions of MF include its use as a remedy for headaches, fever, astringent bowel and as an antidiarrheal compound. MF has many biological activities, including antioxidant, anti-inflammatory, antimicrobial, anti-carcinogenic, and radio-protective activities [4,5,6]. MF has essential and fatty oils, resins, and wax. The concentrations of essential oils in MF are approximately 5–15%, and its major components are terpene hydrocarbons (camphene, *p*-cymene, limonene, myrcene, phellandrene, pinene, sabinene, and terpinene), oxygenated terpenes (geraniol, linalool, and terpineol), and aromatic ethers (myristicin, elemicin, safrole, eugenol, and eugenol derivatives) [7,8]. Safrole is a colorless oil with antibacterial activities. Myristicin exhibits various pharmacological activities, such as anti-inflammatory, antimicrobial, and antiproliferative activities. However, safrole and myristicin, the food-borne alkenyl-benzene structures, pose potential risks to human health as they have been found to induce rodent liver tumors at high doses. This occurrence may be due to 1-hydroxymetabolites, which are converted to carcinogenic 1-sulfoxymetabolites by sulfotransferases (SULTs) [9]. Safrole or 1-hydroxysafrole has been revealed as a carcinogen via the co-administration of the SULT inhibitor pentachlorophenol (PCP) in mice, which resulted in a reduction of hepatic tumor formation upon long-term dietary administration. Therefore, it is recognized as a weak carcinogen by the US government [10]. Several studies have reported that a high content of myristicin is poisonous and results in hallucinatory side effects owing to the two metabolites of myristicin, 5-allyl-1-methoxy-2,3-dihydroxybenzene and 1-hydroxymyristicin. These metabolites were produced in rat liver microsomes and could be identified in rat urine [11].

Nutmeg, along with its use and consumption as a spice, is often incorporated into products to reduce diarrhea, relieve joint and muscle pain, and treat scars. However, there is currently no research on its use for acne treatment. Therefore, the aim of this study was to develop a method to remove the safrole and myristicin of nutmeg and evaluate its application in the treatment of acne.

## 2. Results

### 2.1. Antibacterial Activity of MFE

First, the *M. fragrans* oil (MFO), MFE-M, MFE-H, and MFE-E were tested against *C. acnes* and *S. aureus* by determining the inhibition ratio using the broth dilution method. MFE-M and MFE-E showed antibacterial activities based on the minimum inhibitory concentration (MIC) values of 0.64 and 0.02 mg/mL, respectively. The MIC values of *S. aureus* were 0.64 and 0.16 mg/mL, respectively. MFE-E exhibited significant antibacterial activity against *C. acnes* and *S. aureus*. The inhibition ratios of MFE-E at 100 µg/mL were 114.53% and 56.29%, respectively.

**Figure 1:**
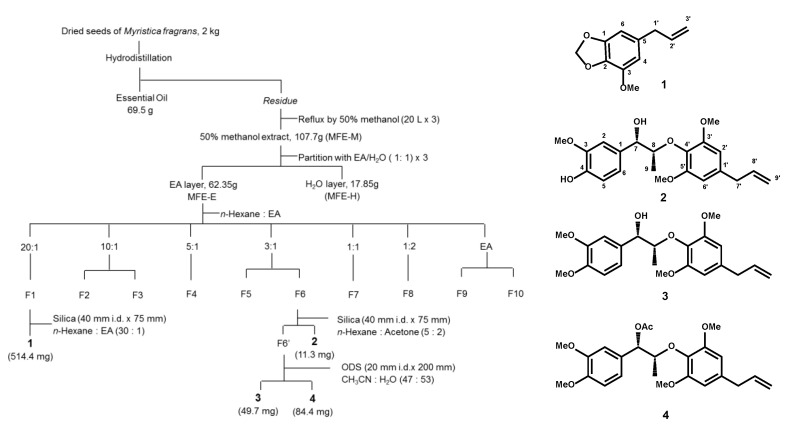
Isolation flowchart of the degreasing of *Myristica fragrans* using antibacterial bioassay-guided fractionation. The structures of myristicin (**1**), (+)-*erythro*-Δ^8′^-7*S*,8*R*- dihydroxy-3,3′,5′-trimethoxy-8-*O*-4′-neolignan (**2**), (+)-*erythro-*Δ^8′^-7-hydroxy-3,4,3′,5′-tetramethoxy- 8-*O*-4′-neolignan (**3**), and e*rythro*-Δ^8′^-7-acetoxy-3,4,3′,5′-tetramethoxy-8-*O*-4′-neolignan (**4**) are shown. F1 to F10 indicated different eluted fractions.

The antibacterial bioassay-guided fractionation flowchart of nutmeg is presented in Figure 1. The following four phytochemicals were identified: one alkenyl-benzene (**1**) and three neolignans (**2**, **3**, and **4**) from nutmeg. The yields of the four phytochemicals were 0.025% (**1**), 0.00005% (**2**), 0.0025% (**3**), and 0.0042% (**4**), respectively. The antibacterial effects were evaluated as previously described. The MICs of the four phytochemicals against *C. acnes* and *S. aureus* are shown in Table 1. Compound 2 displayed a more potent antibacterial activity than that exhibited by other compounds.

### 2.2. NO Inhibitory Effects of MFE-M, MFE-H, and MFE-E

**Figure 2:**
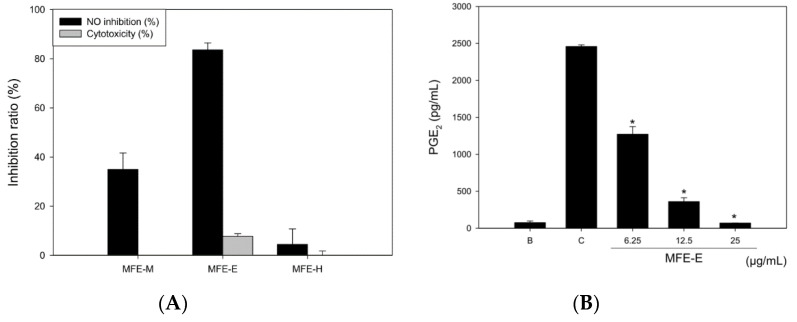
Effects of *M. fragrans* on LPS (500 ng/mL)-stimulated RAW 264.7 cells after 24 h of treatment. NO inhibition percentage of MFE-M, MFE-H, and MFE-E (**A**). PGE_2_ inhibition percentage of MFE-E at various concentrations (6.25–25 µg/mL) (**B**). * *p* < 0.01 compared with Control; B: Blank (without LPS induction), C: Control (LPS induction without MFE-E treatment).

The NO inhibitory effects of MFE-M, MFE-H, and MFE-E were determined in LPS-treated RAW 264.7 cells for 18 h. NO levels were detected in culture medium using the Griess reaction. Survival rates were determined using the MTT assay. The three extracts showed no cytotoxicity at 25 µg/mL. The NO inhibitory rates of MFE-M and MFE-E were 34.93 ± 6.76% and 83.58. ± 2.88%, respectively. MFE-H did not exhibit any NO inhibitory effect (Figure 2A). MFE-E demonstrated dose-dependent PGE_2_ inhibitory effects compared to the other compounds and had an IC_50_ value of 6.25 µg/mL (Figure 2B). Furthermore, the inhibitory effects of the three neolignans were detected by NO and PGE_2_ production in LPS-induced RAW 264.7 cells. The three neolignans at a concentration of 12.5 μg/mL were found to show less than 10% cytotoxicity. The NO inhibitory rates were 61.11% ± 5.19%, 27.78% ± 10.17%, and 43.65% ± 1.37%, respectively. Compound **2** displayed a more potent NO inhibition than the other compounds, and its IC_50_ value was 11.07 μg/mL.

### 2.3. Neolignan Exerted Anti-Inflammatory Effects in RAW 264.7 Cells Treated with Heat-Killed C. acnes

**Figure 3:**
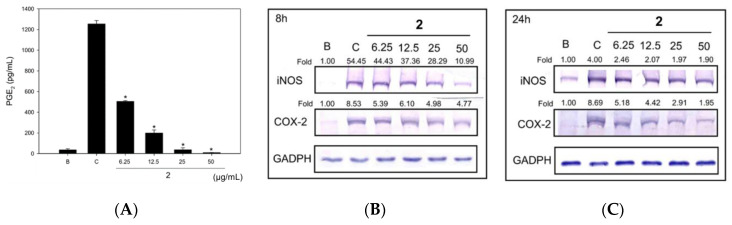
Anti-inflammatory effects of (+)-*Erythro*-(*7S,8R*)-Δ8′,3,3′,5′-trimethoxy-8-*O*-4′-neolignan (**2**) (**A**) against HKC-induced PGE_2_ inhibitory effects on nitric oxide synthase (iNOS) and cyclooxygenase (COX)-2 at 8 h (**B**) and 24 h (**C**). * *p* < 0.01 compared with Control; B: Blank (without HKC induction), C: Control (HKC induction without **2** treatment).

Moreover, in this study, we used heat-killed *C. acnes* (HKC) instead of LPS to simulate the irritation and inflammation caused by *C. acne* infection. Compound **2** at 6.25.50 μg/mL exerted NO inhibitory effects against HKC-induced RAW 264.7 cells. Further, its IC_50_ value was 11.53 μg/mL (Figure 3A). Compound **2** also significantly inhibited PGE_2_ production in HKC-treated RAW 264.7 cells in a dose-dependent manner (Figure 3B). Among the compounds, **2** exhibited good inhibitory effects on iNOS and COX-2 expression at 8 or 24 h (Figure 3).

### 2.4. Quality Control of Myristicin and Safrole Characteristics

**Figure 4:**
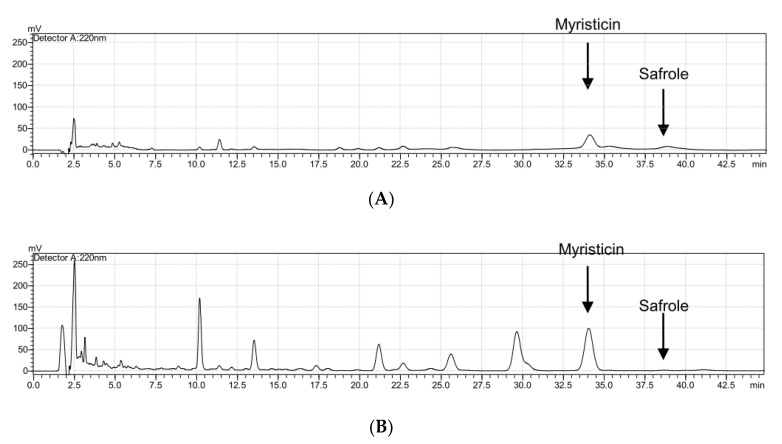
HPLC chromatograms displaying the components of MFO (**A**) and MFE-M (**B**).

Calibration curves were plotted using the peak areas. The calibration curve of myristicin was y = 55,665x − 252,568 and its R square value was R^2^ = 0.9986, while the calibration curve of safrole was y = 13,798x + 50,255 and its R square value was R^2^ = 0.9943. Safrole contained 4.46% and 0.01% MFO and MFE-M, respectively, while myristicin contained 2.62% and 2.49% MFO and MFE-M, respectively (Figure 4).

### 2.5. MFO and MFE-M Did Not Cause Skin Irritation

In this study, 80% of MFO and 0.1 mg/mL and 1 mg/mL of MFE-E were evaluated via a single topical application to Wistar rats. None of the samples resulted in skin irritation in Wistar skin for 24–48 h, thereby indicating the excellent safety of topical treatment (data not shown).

## 3. Discussion

Nutmeg is a tropical evergreen plant that can be used as spices and incorporated into foods and even medicinal products. The spice nutmeg has a distinctive pungent fragrance and a warm, slightly sweet taste. Its essential oil is widely used as condiments and carminatives and can be used to provide scents in soaps and perfumes. An ointment of nutmeg butter has been used as a counterirritant and as a treatment for rheumatism. However, when nutmeg is consumed in large amounts, it has psychoactive effects and is reported to act as a deliriant and hallucinogen. Nutmeg poisoning is rarely fatal but can cause convulsions, palpitations, and pain. Myristicin can cause nerve damage and exerts hallucinogenic effects. The symptoms of overdose include coma, dilated pupils, and convulsions. In the current study, treatment with doses of 0.1% and 0.5% (a carcinogenic dose) for 13 weeks could cause hepatotoxicity [12]. These two components are mainly present in nutmeg essential oil because of their low polarity. Based on the aim of traditional Chinese medicine processing, “Nutmeg must be subjected to degreasing to be ensure safe usage” to attenuate toxicity and to enhance its efficacy. Therefore, by comparing the traditional processing purpose in ancient books and modern scientific research, the same conclusion has been drawn.

To quickly remove the toxic components of nutmeg and increase its efficacy, steam distillation was employed to remove the essential oil, and extraction was performed with 50% methanol. Using high-performance liquid chromatography as an analysis method, the content of safrole was found to be much lower in MFE-M than in MFO. Additionally, only 2.49% of the myristicin remained in the MFE-M. Silica gel column chromatography with the elution solvent, *n*-hexane: Ethyl acetate (EA) = 20:1, was further employed to remove myristicin from MFE-M. Here, we found that the active ingredient, (+)-*erythro*-Δ^8′^-7*S*,8*R*-dihydroxy-3,3,5-trimethoxy-8-*O*-4-neolignan (**2**), exhibited antibacterial activities against *C. acnes* and *S. aureus*, and its anti-inflammatory activities inhibited NO and PGE_2_ production in heat-killed *C. acnes*-treated RAW 264.7 cells. In the present study, we developed a safe and good anti-acne nutmeg extract.

Nutmeg is mainly composed of volatile oils, terpenoids, and lignins. Additionally, other natural lignins isolated from nutmeg have been reported to display antibacterial [13], antioxidant [14], and anti-inflammatory activity [15]. In this experiment, three 8-*O*-4’-neolignans were isolated from nutmeg. To date, there have been no reports of (+)-*erythro*-Δ^8′^-7*S*,8*R*- dihydroxy-3,3,5-trimethoxy-8-*O*-4-neolignan (**2**) and its antibacterial and anti-inflammatory activities. According to its structure-activity relationship (SAR), the -OH group on C-4 may be an important functional group in its anti-acne activity. This study is the first to explore the anti-acne effects of this type of 8-*O*-4’-neolignans.

In the treatment of acne, topical treatment is safer than oral treatment because of its lower side effects. Accordingly, the MFO and MFE-E extracts were further evaluated via a single topical application in Wistar rats. The results showed that high doses of MFO resulted in nonirritating effects. Furthermore, the high concentration of MFE-E did not cause toxicity in the skin. Collectively, our findings indicate that the essential oil of nutmeg is a common source of spices but exhibits different toxicities and side effects. To our knowledge, this study is the first to evaluate the anti-acne effect of degreasing nutmeg. MFE-E could thus be used as a source to develop external anti-acne medicines and cosmetics and may function as additives in cleaning products.

## 4. Materials and Methods

### 4.1. General

Myristicin (≥98.5%, Merck code 09237), safrole (≥98.5%, Supelco code 08010), dimethyl sulfoxide (DMSO), lipopolysaccharide (LPS), 3-(4.5-dimethylthiazol-2-yl)-2.5-diphenyltetrazolium bromide (MTT), and other chemicals were purchased from Sigma (St. Louis, MO, USA). Dulbecco’s modified Eagle medium (DMEM), fetal bovine serum (FBS), antibiotics, and glutamine were purchased from GIBCO BRL (Grand Island, NY, USA). The bacterial culture equipment, including an anaerobic atmosphere by MGC AnaeroPack-Jar and MGC AnaeroPack-Anaero, were purchased from Mitsubishi Gas Chemical (Tokyo, Japan). Blood agar plates (BAPs), Bacto^TM^ tryptic soy agar (TSA), and BactoTM tryptic soy broth (TSB) were purchased from Difco (Detroit, MI, USA).

### 4.2. Nuclear Magnetic Resonance (NMR) and Mass Spectrometry (MS) Instruments

^1^H (500 MHz) and ^13^C NMR (126 MHz) spectra were generated on a Bruker DRX 500 spectrometric system using CDCl_3_ as the solvent, and chemical shifts are given in δ (ppm) values. Two-dimensional NMR spectra (^1^H–^1^H COSY, HMQC, HMBC, NOESY) were obtained using a Bruker DRX 500 spectrometer using standard Bruker pulse sequences (Bruker, Rheinstetten, Germany). Electrospray ionization mass spectra (ESI-MS) were analyzed using the SCIEX API-4000 Q system (Sciex Division of MDS, Toronto, ON, Canada).

### 4.3. Extraction and Isolation of M. fragrans

*M. fragrans* was purchased from Sun Ten Pharmaceutical (New Taipei City, Taiwan) and authenticated by the nonprofit organization Brion Research Institute of Taiwan (New Taipei City, Taiwan). Voucher specimens (MF-01) were identified and deposited at the School of Pharmacy, Taipei Medical University. Dried *M. fragrans* (2000 g) was pulverized and filtered through a 20# mesh. *M. fragrans* oil (MFO) was obtained through hydrodistillation of the dried *M. fragrans* in a Clevenger-type apparatus for 2 h, partition with ether, and air-dried. The MFO was stored at 4 °C until analysis (69.5 g). The *M. fragrans* residue was extracted using 50% aqueous methanol (20 L × 3) at 65 °C for 2 h. The MeOH extract was evaporated under reduced pressure (~40 °C) to produce residual MFE-M (107.7 g). The MFE-M was dissolved in ethyl acetate (MFE-E) and partitioned with distilled water (MFE-H). The EtOAc layer was subjected to column chromatography on silica gel using n-hexane, an n-hexane and EtOAc mixture of increasing polarity, and pure acetone to yield 10 fractions. Fraction 1 (*n*-hexane: EA = 20:1) was further purified with silica gel to obtain myristicin (**1**). Fraction 6 (*n*-hexane:EA = 3:1) was separated by normal-phase HPLC to yield (+)-*erythro*-Δ^8′^-7*S*,8*R*-dihydroxy-3,3,5′-trimethoxy-8-*O*-4′-neolignan (**2**) and was applied to an ODS column eluted with 0.05% trifluoroacetic acid-CH_3_CN (47: 53) to obtain (+)-*erythro-*Δ^8’^-7-hydroxy- 3,4,3’,5’-tetramethoxy-8-*O*-4’-neolignan (**3**) and e*rythro*-Δ^8′^-7-acetoxy-3,4,3′,5′-tetramethoxy-8-*O*-4′-neolignan (**4**) (Figure 1).

Myristicin (**1**) was isolated as a transparent oil. ESI(+)-MS (*m/z*) 193 [M + H]. ^1^H NMR (500 MHz, CDCl_3_) δ: 6.39 (1H, br s, H-6), 6.35 (1H, br s, H-4), 5.92 (2H, s, O-CH_2_-O), 5.90 (1H, m, H-2′), 5.10-5.05 (2H, m, H-3′), 3.89 (3H, s, OCH_3_), 3.29 (2H, br d, *J* = 6.5 Hz, H-1′). ^13^C NMR (125 MHz, CDCl_3_) δ: 148.8 (C-2), 143.5 (C-1), 137.3 (C-3), 134.6 (C-2′), 133.5 (C-5), 115.8 (C-3′), 107.7 (C-4), 102.7 (C-6), 101.2 (O-CH_2_-O), 56.5 (OCH_3_), 40.2 (C-1′) [16].

(+)-*erythro*-Δ^8′^-7*S*,8*R*-dihydroxy-3,3,5-trimethoxy-8-*O*-4-neolignan (**2**) was isolated as a transparent oil. ESI(-)-MS (*m/z*) 373 [M-H]^−^. ^1^H NMR (500MHz, CDCl_3_) δ: 6.93 (1H, d, *J* = 1.6 Hz, H-2), 6.72 (1H, d, *J* = 8.2 Hz, H-5), 6.68 (1H, dd, *J* = 8.2, 1.6 Hz, H-6), 6.52 (2H, br s, H-2′ and H-6′), 5.97 (1H, ddt, *J* = 17.1, 10.4, 6.7 Hz, H-8′), 5.12 (1H, dd, *J* = 17.1, 1.8 Hz, H-9′a), 5.05 (1H, dd, *J* = 10.4, 1.8 Hz, H-9′b), 4.74 (1H, d, *J* = 3.7 Hz, H-7), 4.27 (1H, qd, *J* = 6.4, 3.7 Hz, H-8), 3.81 (9H, s, 3-OMe, 3′-OMe and 5′-OMe), 3.34 (2H, d, *J* = 6.7 Hz, H-7′), 1.09 (3H, d, *J* = 6.4 Hz, H-9). ^13^C NMR (125 MHz, CDCl_3_) δ: 154.7 (C-3′ and C-5′), 148.7 (C-4), 146.7 (C-3), 138.8 (C-8′), 137.7 (C-1′), 134.8 (C-4′), 133.5 (C-1), 120.1 (C-6), 116.1 (C-9′), 115.8 (C-5), 111.1 (C-2), 106.9 (C-2′ and C-6′), 83.8 (C-8), 75.5 (C-7), 56.6 (3′-OMe and 5′-OMe), 56.4 (3-OMe), 41.4 (C-7′), 13.8 (C-9) [17].

(+)-*erythro-*Δ^8’^-7-Hydroxy-3,4,3’,5’-tetramethoxy-8-*O*-4’-neolignan (**3**) was isolated as a transparent oil. ESI(+)-MS (*m/z*) 411 [M + Na]^+^. ^1^H NMR (500MHz, CDCl_3_) δ: 6.95 (1H, d, *J* = 1.6 Hz, H-2), 6.84 (1H, d, *J* = 8.2 Hz, H-5), 6.77 (1H, dd, *J* = 8.2, 1.6 Hz, H-6), 6.46 (2H, br s, H-2′ and H-6′), 5.96 (1H, ddt, *J* = 17.1, 9.8, 6.7 Hz, H-8′), 5.12 (1H, dd, *J* = 17.1, 1.8 Hz, H-9′a), 5.09 (1H, dd, *J* = 10.0, 1.8 Hz, H-9′b), 4.80 (1H, d, *J* = 3.1 Hz, H-7), 4.33 (1H, qd, *J* = 6.7, 3.1 Hz, H-8), 3.86 (3H, s, 4-OMe), 3.85 (3H, s, 3-OMe), 3.83 (6H, s, 3′-OMe and 5′-OMe), 3.35 (2H, d, *J* = 6.7 Hz, H-7′), 1.10 (3H, d, *J* = 6.1 Hz, H-9). ^13^C NMR (125 MHz, CDCl_3_) δ: 153.5 (C-3′ and C-5′), 148.8 (C-4), 147.9 (C-3), 137.1 (C-8′), 136.1 (C-4′), 133.0 (C-1′), 132.7 (C-1), 118.1 (C-6), 116.2 (C-9′), 110.8 (C-2), 109.3 (C-5), 105.5 (C-2′ and C-6′), 82.3 (C-8), 72.8 (C-7), 56.1 (3′-OMe and 5′-OMe), 55.9 (3-OMe and 4-OMe), 40.6 (C-7′), 12.8 (C-9) [17].

*Erythro*-Δ8-7-Acetoxy-3,4,3,5-tetramethoxy-8-O-4- neolignan (**4**) was isolated as a transparent oil. ESI(+)-MS (*m/z*) 453 [M + Na]^+^. ^1^H NMR (500 MHz, CDCl_3_) δ: 6.88 (1H, d, *J* = 1.7 Hz, H-2), 6.80 (1H, d, *J* = 8.2 Hz, H-5), 6.84 (1H, dd, *J* = 8.2, 1.7 Hz, H-6), 6.39 (2H, br s, H-2′ and H-6′), 5.96 (1H, ddt, *J* = 17.1, 10.4, 6.6 Hz, H-8′), 5.86 (1H, d, *J* = 3.4 Hz, H-7), 5.11 (1H, dd, *J* = 17.9, 1.8 Hz, H-9′a), 5.08 (1H, dd, *J* = 10.4, 1.8 Hz, H-9′b), 4.43 (1H, qd, *J* = 6.7, 3.7 Hz, H-8), 3.86 (3H, s, 4-OMe), 3.85 (3H, s, 3-OMe), 3.77 (6H, s, 3′-OMe and 5′-OMe), 3.33 (2H, d, *J* = 6.6 Hz, H-7′), 2.17 (3H, s, 7-OAc), 1.28 (3H, d, *J* = 6.7 Hz, H-9). ). ^13^C NMR (125 MHz, CDCl_3_) δ: 170.2 (7-OAc),153.4 (C-3′ and C-5′), 148.7 (C-4),148.5 (C-3), 137.2 (C-8′), 135.7 (C-4′), 133.8 (C-1′), 130.6 (C-1), 119.3 (C-6), 116.0 (C-9′), 110.8 (C-2), 110.3 (C-5), 105.6 (C-2′ and C-6′), 80.1 (C-8), 76.4 (C-7), 56.0 (3′-OMe and 5′-OMe), 55.9 (3-OMe and 4-OMe), 40.5 (C-7′), 21.2 (7-OAc), 14.5 (C-9) [6].

### 4.4. Antibacterial Activities against Cutibacterium Acnes and Staphylococcus aureus

The strains of *C. acnes* (BCRC10723, isolated from facial acne) and *S. aureus* (BCRC 10781, ATCC 25923) were obtained from the Bioresource Collection and Research Center (Hsinchu, Taiwan). *C. acnes* were cultured in BAP, BactoTM TSB, and BactoTM TSA in an anaerobic condition using MGC AnaeroPack-Anaero and MGC AnaeroPack-Jar. *S. aureus* was cultured in BactoTM TSB and BactoTM TSA.

The experimental protocol followed a previous report with modifications [18]. MFO, MFE-M, MFE-E, MFE-H, and compounds **1**, **2**, **3**, and **4** were tested against *C. acnes* by determining the MIC values through the broth-dilution method [19]. A freshly grown culture was diluted with BactoTM TSB, and 1 mL of the prepared bacteria (4 × 10^8^ CFU/mL). Triton X-100 was used as a positive control. Samples were diluted in sterile broth and then mixed with broth inoculated with *C. acnes*. Dilutions were prepared at 10-fold the desired final concentration. The test concentrations of MFE-M, MFE-E, and MFE-H were 0.02.10.24 mg/mL. The test concentrations of compounds **1**, **2**, **3** and **4** were 3.12–100 µg/mL. Test samples were incubated under anaerobic conditions at 37 °C for 24 h until visible growth of the test microorganisms was observed in the control. The O.D. value was measured using an ELISA reader at 600 nm. The inhibition ratio was compared to that of Triton X-100. The MIC experiment was repeated in triplicate. Moreover, *S. aureus* (0.5 × 10^8^ CFU/mL) was prepared. The experimental protocol was the same as that previously described, with some modifications. The antimicrobial activity was expressed as the O.D. value of the inhibition rate against the test microorganisms: Ratio (%) = (O.D. of sample/O.D. Triton X-100) × 100.

### 4.5. Anti-Inflammatory Activity against LPS-Treated RAW 264.7 Cells

The anti-inflammatory activities of the compounds were evaluated in RAW 264.7 cells cultured in DMEM with 10% FBS, 1% L-glutamine, and 1% penicillin-streptomycin, and maintained at 37 °C in a 5% CO_2_ atmosphere. RAW 264.7 cells (4.0 × 10^5^ cells/mL) were seeded in 96-well plates and co-treated with LPS (500 ng/mL) and the test samples. After 24 h, NO production was determined with Griess reagent, and the absorption was detected at 530 nm. The inhibitory percentage of NO production was presented as an anti-inflammatory activity. Prostaglandin E_2_ (PGE_2_) concentrations in the cell culture media were determined using a PGE_2_ enzyme-linked immunosorbent assay (ELISA) kit (Enzo Life Sciences, Farmingdale, NY, USA). The survival rate of RAW 264.7 cells was detected by an MTT assay.

### 4.6. Anti-Inflammatory Activity against Heat-Killed C. acnes-Treated RAW 264.7 Cells

*C. acnes* (4 × 10^8^ cfu/mL) was heated to 80 °C for 30 min to kill the bacteria. HKC was freeze-dried as a powder and maintained at 4 °C until use. RAW 264.7 cells were cultured in DMEM with 10% FBS, 1% penicillin-streptomycin, and 1% l-glutamine at 37 °C in a 5% CO_2_ atmosphere. RAW 264.7 cells (4 × 10^5^ cells/mL) were seeded in six-well plates and co-treated with HKC (300 μg/mL) and test samples. NO and PGE_2_ production were determined according to previously described methods [20]. Whole-cell lysates were prepared via lysis with radioimmunoprecipitation assay buffer. The proteins in the lysate (30 μg) were denatured in sodium dodecyl sulfate-polyacrylamide gel electrophoresis (SDS-PAGE) sample buffer and analyzed by 10% SDS-PAGE. Gels were transferred to nitrocellulose membranes and probed with primary iNOS (clone N-20), COX-2 (clone C-20), and glyceraldehyde-3-phosphate dehydrogenase (GAPDH; clone 6C5) antibodies from Santa Cruz Biotechnology (Santa Cruz, CA, USA). Membranes were visualized with a BCIP/NBT kit (Gibco BRL, Grand Island, NY, USA). The level of protein expression was quantified using an Azure Biosystem C300 Imaging System. GAPDH expression was used as an internal control to compare the protein expression of iNOS and COX-2.

### 4.7. Quality Control of Myristicin and Safrole

The HPLC apparatus included an SCL-10Avp system *controller*, an LC-10ATvp liquid chromatographic pump, an SPD-M10A diode array detector, an SIL-10Avp auto-injector, a CTO-10A column oven, FCV-10Avp flow-channel selection valves (Shimadzu, Tokyo, Japan), and an ERC-3415 degasser (ERC, Altegolfsheim, Regensburg, Germany). The stationary phase comprised a Purospher^®^ STAR RP-18e reverse-phase column (4 mm i.d. × 250 mm, 5 μm, Merck, Kenilworth, NJ, USA), and the mobile phase system included 0.05% trifluoroacetic acid : CH_3_CN isocratic at a ratio of 40:60. The flow rate was 1 mL/min, and the column oven temperature was maintained at 40 °C. The ultraviolet wavelength was set to 220 nm to detect myristicin and safrole in MFO and MFE-M. The concentration range of myristicin and safrole was 0.78 to 1000 μg/mL.

### 4.8. Safety Control of Myristicin and Safrole

The skin toxicity of MFO and MFE-E was evaluated by a single topical application in Wistar rats [21]. The following animal experiments were conducted according to the Ethical Regulations on Animal Research of Taipei Medical University (approval no. LAC-100-0030). Female Wistar rats (nulliparous, non-pregnant), aged 8–10 weeks and weighing approximately 200–220 g, were purchased from BioLASCO in Taiwan. Rats were maintained at 25 ± 1 °C with food and water *ad libitum* and maintained on a 12-h light/dark cycle. At 24 h before the test, the backs of the rats were shaved free of hair (2.5 × 2.5 cm at each site) and checked for any abnormalities (integrity and allergies). An appropriate concentration of MFO and MFE-E (50 μL per sample) was smeared onto the shaved back of a Wistar rat. The patches were secured for a 4-h exposure period and removed with distilled water. At 24 and 48 h after the application of MFO and MFE-E, skin irritation was observed, and the extent of an evident skin allergic reaction was evaluated for each test animal. An acute primary irritation test was performed following the classic Draize test.

### 4.9. Statistical Analysis

The data are presented as the mean and standard deviation (S.D.). Significance was calculated using Student’s *t*-test. Differences were considered significant at *p* < 0.05.

## Figures and Tables

**Table 1:** Antibacterial activity of myristicin and three neolignans.

Microorganisms	MIC µg/mL			
	1	2	3	4
*Cutibacterium acnes*	>100	6.25	100	25
*Staphylococcus aureus*	12.5	3.12	6.25	12.5
